# The effect of time of measurement on the discriminant ability for mortality in trauma of a pre-hospital shock index multiplied by age and divided by the Glasgow Coma Score: a registry study

**DOI:** 10.1186/s12873-022-00749-8

**Published:** 2022-11-30

**Authors:** Mikael Laaksonen, Johannes Björkman, Timo Iirola, Lasse Raatiniemi, Jouni Nurmi

**Affiliations:** 1 Department of Perioperative Services, Intensive Care Medicine and Pain Management, Turku University Hospital and University of Turku, Turku, Finland; 2 FinnHEMS Research and Development Unit, Vantaa, Finland; 3 Emergency Medical Services, Turku University Hospital and University of Turku, Turku, Finland; 4 Centre for Emergency Medical Services, Oulu University Hospital, Oulu, Finland; 5 Emergency Medicine and Services, Helsinki University Hospital and University of Helsinki, Helsinki, Finland

**Keywords:** Emergency medical services, Critical care, Shock index

## Abstract

**Background:**

The shock index (SI) and its derivatives have been shown to predict mortality in severely injured patients, both in pre-hospital and in-hospital settings. However, the impact of the time of measurement on the discriminative ability of the pre-hospital SI is unknown. The aim of this study was to evaluate whether the time of measurement influences the discriminative ability of the SI multiplied by age (SIA) and divided by the Glasgow Coma Score (SIA/G).

**Methods:**

Registry data were obtained from the national helicopter emergency medical services (HEMS) on trauma patients aged ≥ 18 years. The SI values were calculated based on the first measured vitals of the trauma patients by the HEMS unit. The discriminative ability of the SIA/G, with 30-day mortality as the endpoint, was evaluated according to different delay times (0 − 19, 20 − 39 and ≥ 40 min) from the initial incident. Sub-group analyses were performed for trauma patients without a traumatic brain injury (TBI), patients with an isolated TBI and patients with polytrauma, including a TBI.

**Results:**

In total, 3,497 patients were included in the study. The SIA/G was higher in non-survivors (median 7.8 [interquartile range 4.7–12.3] vs. 2.4 [1.7–3.6], *P* < 0.001). The overall area under the receiver operator characteristic curve (AUROC) for the SIA/G was 0.87 (95% CI: 0.85–0.89). The AUROC for the SIA/G was similar in the short (0.88, 95% CI: 0.85–0.91), intermediate (0.86, 95% CI: 0.84–0.89) and long (0.86, 95% CI: 0.82–0.89) measurement delay groups. The findings were similar in the three trauma sub-groups.

**Conclusions:**

The discriminative ability of the SIA/G in predicting 30-day mortality was not significantly affected by the measurement time of the index in the pre-hospital setting. The SIA/G is a simple and reliable tool for assessing the risk of mortality among severely injured patients in the pre-hospital setting.

**Supplementary Information:**

The online version contains supplementary material available at 10.1186/s12873-022-00749-8.

## Background

Trauma is one of the leading causes of death and disability worldwide, with a considerable portion of the injured being young adults [1]. Therefore, it is vital to be able to identify critically ill, deteriorating patients to provide optimal early treatment. A simple instrument to evaluate mortality and facilitate resource allocation throughout the chain-of-treatment would be highly useful, particularly in the pre-hospital setting.

The shock index (SI), originally proposed by Allgöwer et al. in 1967, was developed as a simple tool for detection of circulatory collapse in haemodynamically unstable patients and has been shown to have predictive value in trauma patient mortality [2, 3]. The SI, which is determined by the heart rate divided by the systolic blood pressure (mmHg), has since been used in a variety of settings. A normal SI is categorized as 0.5–0.7, with an SI above 1 indicating uncompensated haemodynamic shock, which is accompanied by increased morbidity and mortality [3–6].

Several modifications of the SI have been proposed to increase its prognostic value. Earlier studies increased the predictive value of the SI by incorporating variables, such as age and the Glasgow Coma Score (GCS), into models [4] Further, the functionality of the SI has been assessed using a reverse SI (rSI), on the premise that clinicians typically view uncompensated circulatory shock as systolic blood pressure lower than the heart rate, not the other way around [7, 8]. A model incorporating both age and GCS into the SI, defined as the SI multiplied by age and divided by the GCS (SIA/G), has been shown to be more accurate than the SI, GCS or age alone, especially in elderly patients [3, 4].

Other potential variables affect the predictive value of the SI and its variations. In real life settings, for example, the delay in reaching a trauma patient varies greatly due to limitations in geography, infrastructure, and availability of resources. At present, the SI is considered a dynamic variable, with conflicting results, and the accuracy of the SI in identifying high-risk patients according to the time from an accident to the measurement of the SI remains unclear [5, 9, 10]. For appropriate use of the SI in decision making, including in trauma centres, this should be clarified.

The aim of this study was to evaluate whether the time of measurement of the SIA/G influences its discriminative ability in terms of 30-day mortality.

## Methods

This was a retrospective cohort study combining data from national registries. The discriminative ability of the SIA/G was evaluated in trauma patients treated by the helicopter emergency medical services (HEMS) with different delay times from the incident. The study did not affect patient treatment and therefore patient consent was not required nor acquired. The Ethical Committee of Helsinki University Hospital approved the waiver for the need for informed consent due to only registry data was used. STROBE (The Strengthening the Reporting of Observational Studies in Epidemiology) guidelines were followed in reporting of the study [11].

The study protocol was approved by the ethics committee of Helsinki University Hospital (HUS/3115/2019 §194). The hospital districts responsible for the HEMS (Oulu University Hospital: 200/2019 2.7.2019; Helsinki University Hospital: HUS/280/2019 9.7.2019; Turku University Hospital: J30/19 4.8.2019; the Hospital District of Lapland: 32/2019 22.8.2019; Kuopio University Hospital: RPL 102/2019 22.8.2019; and Tampere University Hospital: RTL-R19580 2.9.2019), Population Register Centre (VRK/5613/2019–3 1.11.2019) and Finnish institute for health and welfare (VRK/5613/2019–3 1.11.2019) responsible for the hospital discharge registry also approved the protocol. All methods used are in accordance with the Declaration of Helsinki.

### Setting

The patients were treated by the national HEMS personnel. The HEMS consist of five units staffed by a physician and one unit staffed by a paramedic with specific training in pre-hospital critical care. The HEMS is part of public health care in Finland and free of charge for patients.

HEMS units are dispatched by emergency response centres in response to emergency calls. They are dispatched simultaneously with the emergency medical service (EMS) units based on pre-determined criteria to provide pre-hospital critical care and transport/escort patients directly to a tertiary hospital, if appropriate. HEMS units can also be dispatched on request from the EMS unit at a scene. Each HEMS mission is recorded in a national database by the physician or paramedic on call. The national HEMS and database have been described recently in detail [12].

### Participants

We included all trauma patients over 18 years who were treated by the national HEMS from January 1, 2012 to December 31, 2018. Patients with a corrupt or missing personal identification code, as given by the Finnish Population Information System, were excluded, as this was used to identify and combine data from the registries.

### Variables

The exposure studied was the SIA/G, and the primary outcome measurement was 30-day survival. The SI was calculated from the vital signs measured upon patient contact by the HEMS unit. SIA/G was calculated in our study as follows: ((HR/SBP) x Age) / GCS. The initial GCS as evaluated by the HEMS unit was incorporated into the SIA/G. The method of measuring vital signs was not controlled and included different defibrillator and monitoring devices used by EMS units. The SI was calculated from the vital signs measured upon patient contact by the HEMS unit. No serial or further SI measurements were done or analyzed during transport to or at the receiving hospital.

For evaluation of the effect of the delay time from the incident to the recording of the SIA/G on the discriminant ability of the index, the patients were divided into three groups according to the elapsed time between the emergency call and HEMS unit arrival on scene, I.E. first measurement of SI by a HEMS unit. The delay time was then divided into three groups: 0 − 19, 20 − 39 and ≥ 40 min.

The need for airway management during the pre-hospital phase was included in the analyses, as this has been shown to be strongly associated with subsequent mortality in this population [13]. In this study, tracheal intubation or the placement of a supraglottic airway device was considered advanced airway management.

ICD-10 based Injury Severity Scores (ICISS) were created using hospital discharge diagnoses [14].

### Data sources

Data were combined from three registries: the national HEMS database, the national hospital discharge registry and the Population Information System. The national HEMS database has been used since the launch of the national HEMS system in 2012. The database includes the variables according to the international consensus-based recommendations on data collection in physician-staffed pre-hospital care and pre-hospital advanced airway management [15]. Data entry in the hospital discharge registry is mandatory for all hospitals in the country at the time of hospital admission. Hospital-related data included in the registry include data on the length of stay, diagnoses, and procedures.

The Population Information System includes data on Finnish citizens and foreign citizens who are residents in Finland. These data include the date of birth, place of residence and the time of death. In Finland, a personal identity code is automatically issued to each resident by the Finnish Population Information System. This code was used to identify and combine data from the different registries.

### Sample size

No power calculation was performed, as all data available at the time of formation of the study dataset were included.

### Statistical analysis

The distribution of the variables was tested using the D’agostino − Pearson omnibus normality test. As all the variables were non-normally distributed, non-parametric tests were used in comparisons, and the data were reported as medians (interquartile range). The discriminant ability of the SIA/G was evaluated by visualization of receiver operator characteristic curves (ROCs) and calculating the area under the ROC (AUROC), with 95% confidence intervals (95% CIs). Sub-groups of traumatic brain injury (TBI) patients were evaluated. The ROCs were compared using the following equation: $Z=\frac{\left|{Area}_{1}-{Area}_{2}\right|}{\sqrt{{SE}_{Area1}^{2}+{SE}_{Area2}^{2}}}$. The two-tailed *P* values were consequently calculated from Z scores using Microsoft Excel. The TBI classification was based on the hospital discharge diagnosis. A TBI was classified according to an ICD-10 diagnosis of an intracranial injury. The patients were divided into three TBI subgroups: trauma without a TBI, an isolated TBI or polytrauma, including a TBI. A detailed list of the diagnoses is presented in Additional file 1: Appendix 1. The statistical analyses were performed using SPSS 27 (IBM Corporation, Armonk, NY, USA).

## Results

**Fig. 1:**
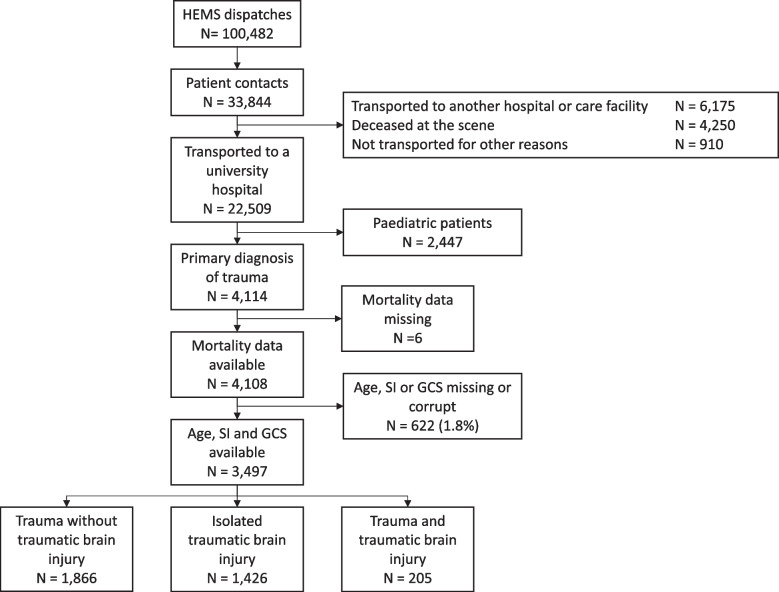
Patient selection flow chart. HEMS, helicopter emergency medical services; SI, shock index; GCS, Glasgow Coma Score

In total, 3,497 patients were included in the analyses (Fig. 1). Of these, 1,866 (51%) patients had a trauma without a TBI, 1,426 (43%) patients had an isolated TBI, and 205 (6%) patients had polytrauma, including a TBI. In terms of HEMS delay times, these were 0 − 19, 20 − 39 and ≥ 40 min in 1,402, 1,478 and 617 of cases, respectively. The characteristics of the patients are presented in Table 1. Mortality was higher and airway management more frequently performed in the patients with longer HEMS delay times (Table 2). In the study population, the crude 30-day mortality was 14.0% (488/3,497).

**Table 1:** Patient characteristics

	All patients *N* = 3,497		Trauma without TBI *n* = 1,866		Isolated TBI *n* = 1,426		Multi-trauma with TBI *n* = 205	
Age (years)	49.2 (31.6 − 65.2)		46.2 (30.3 − 61.2)		55.1 (34.5 − 70.1)		48.1 (31.6 − 60.8)	
Sex (male)	2,536 (72.5)		1389 (74.4)		984 (69.0)		163 (79.5)	
Heart rate (beats per minute)	90 (78 − 103)		90 (79 − 102)		89 (75 − 101)		98 (77 − 110)	
Systolic blood pressure (mmHg)	132 (114 − 151)		129 (110 − 145)		140 (120 − 161)		125 (111 − 152)	
GCS	14 (7 − 15)		15 (14 − 15)		8 (4 − 13)		9 (4 − 14)	
Advanced airway management	1075 (30.7)		223 (12.0)		733 (51.4)		118 (57.6)	
HEMS delay, minutes	22 (15 − 34)		23 (16 − 33)		22 (15 − 35)		25 (16 − 38)	

Values are median (interquartile range) or number (proportion)

*GCS* Glasgow Coma Score, *HEMS* Helicopter emergency medical services, *TBI* Traumatic brain injury

**Table 2:** Comparison of the sub-groups according to delay times from the emergency call to patient treatment by the helicopter emergency medical services (HEMS)

	0 − 19 min *n* = 1,402		20 − 39 min *n* = 1,478		≥ 40 min *n* = 617		*P* value
Age (years)	50.0 (31.2 − 64.5)		50.3 (32.6 − 66.1)		49.1 (30.5 − 64.7)		0.194
Sex (male)	1,005 (71.7)		1,072 (72.7)		459 (74.4)		0.454
Heart rate (beats per minute)	90 (79 − 105)		90 (78 − 102)		88 (74 − 102)		0.002
Systolic blood pressure (mmHg)	134 (118 − 154)		130 (112 − 150)		130 (110 − 150)		0.002
GCS	14 (7 − 15)		14 (7 − 15)		12 (5 − 15)		< 0.001
Advanced airway management	354 (25.2)		456 (31.0)		266 (43.6)		< 0.001
SIA/G	2.6 (1.8 − 4.4)		2.7 (1.8 − 4.6)		2.8 (1.8 − 5.2)		0.072
Death within 30 days	173 (12.3)		213 (14.5)		102 (16.6)		0.033
ICISS	0.91 (0.80 − 0.98)		0.91 (0.80 − 0.97)		0.85 (0.74 − 0.96)		< 0.001

*GCS* Glasgow Coma Score, *SIA/G* Shock index multiplied by age and divided by the Glasgow Coma Score, *ICISS* ICD-10 based Injury Severity Score

The SIA/G was 2.2 (1.5 − 3.1) in patients without a TBI, 3.9 (2.3 − 7.4) in patients with an isolated TBI and 3.6 (2.3 − 6.7) in polytrauma patients with a TBI. The SIA/G was significantly higher in patients that died within 30 days (7.8 [4.7 − 12.3] vs. 2.4 [1.7 − 3.6], *P* < 0.001).

The overall AUROC of the SIA/G for predicting 30-day mortality was 0.87 (95% CI: 0.85- 0.89). The AUROC in the patients without a TBI was 0.83 (95% CI: 0.78–0.88). It was 0.82 (95% CI: 0.80–0.85) in those with an isolated TBI and 0.84 (95% CI: 0.76–0.92) in multi-trauma patients with a TBI.

**Fig. 2:**
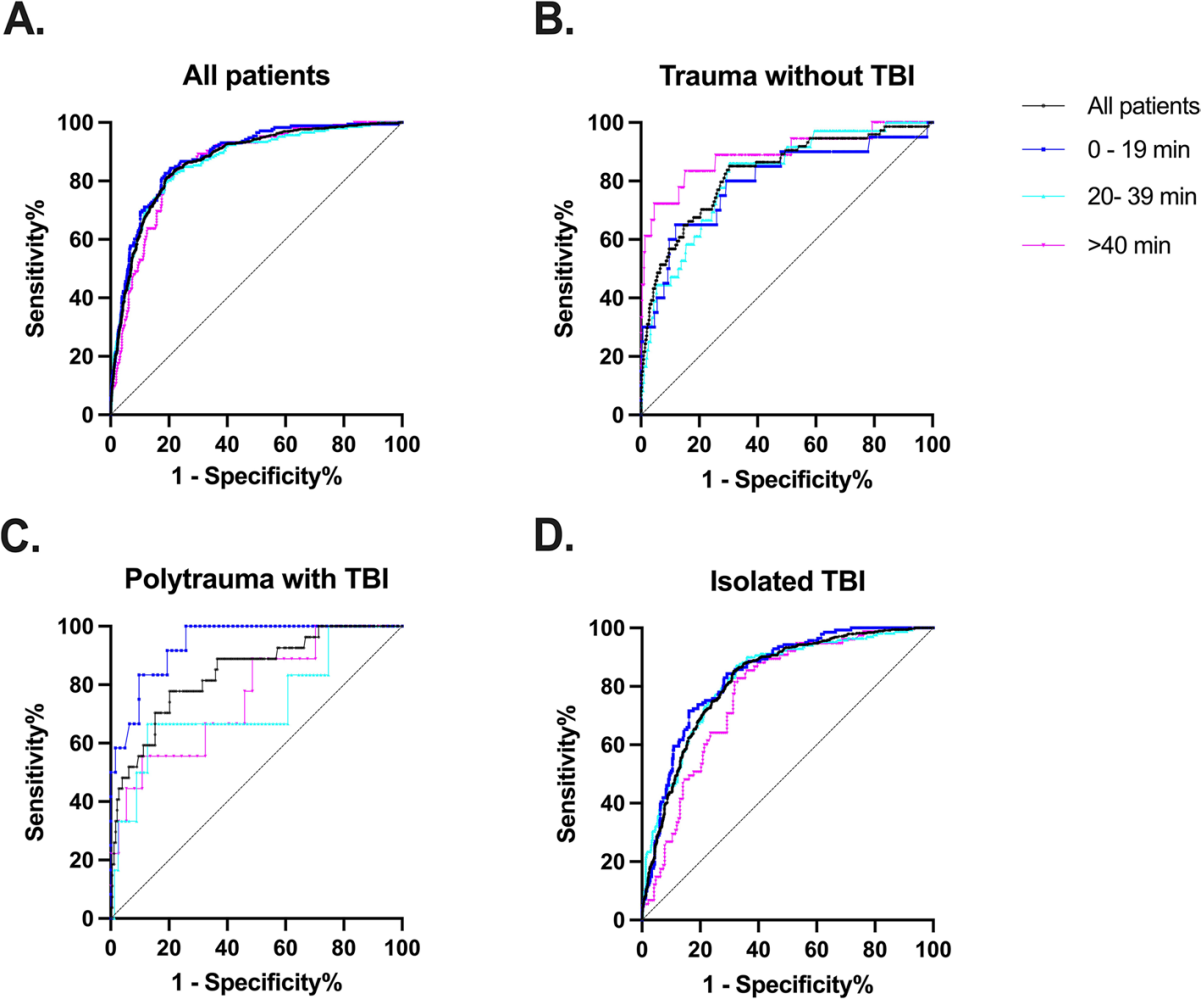
**A − D** Receiver operator characteristic curves (ROCs) for the shock index multiplied by age and divided by the Glasgow Coma Score (SIA/G) for different delay times after an emergency call in predicting 30-day mortality. TBI, traumatic brain injury

**Fig. 3:**
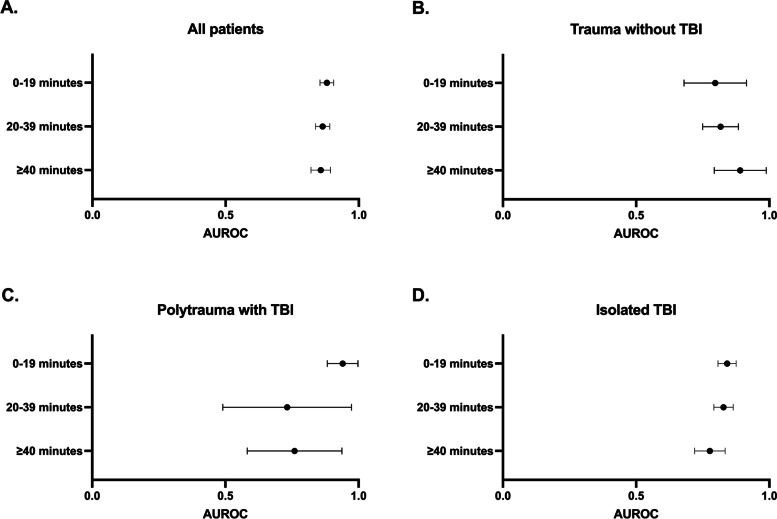
**A − D** Area under the receiver operator characteristic curves (ROCs), with 95% confidence intervals (CIs) for the shock index multiplied by age and divided by the Glasgow Coma Score (SIA/G) in the prediction of 30-day mortality in trauma patients treated by the helicopter emergency medical services (HEMS) unit according to delays in SIA/G measurement times. The AUROC CIs presented in Fig. 3 show no statistically significant difference between the delay groups. Please refer to Additional file 3: Appendix 3 for numerical results

There was no discernible difference for the ROCs of the SIA/G for prediction of 30-day mortality between the groups with different time delays (Fig. 2). The AUROC CIs are presented in Fig. 3, showing no statistically significant difference between the delay groups. Additional file 2: Appendix 2 displays Z scores and *P* values for different patient groups.

## Discussion

This study demonstrated that the discriminative ability of the pre-hospital SIA/G was not affected by the time at which the index was first measured. To our knowledge, this is the first study to report the impact of the time of measurement on the utility of a pre-hospital age-adjusted SI incorporating the GCS. Furthermore, we found that the SIA/G was a good differentiator of severely injured patients with a high risk of death.

Critically injured trauma patients require a streamlined treatment protocol and advance preparation in the pre-hospital setting for the emergency department to ensure optimal treatment and resource allocation. The SI and its derivatives have been shown to be simple tools for predicting the need for the highest level of trauma-team activation, blood transfusions and mechanical ventilation and mortality in severely injured patients, with a single measurement in the in-hospital setting being predictive of mortality [3–6, 16–19]. The present findings on the SIA/G could be used to develop HEMS cancellation and secondary dispatch criteria.

To our knowledge, no previous studies have researched the impact of the pre-hospital time of measurement of the SIA/G on its discriminative ability regarding mortality. Overall, in our study, the discriminative ability of SIA/G regarding mortality remained unchanged, regardless of the time of the measurement. Previous studies where the SI was utilized as a dynamic variable reported somewhat contradictory results. According to these studies, an unchanged or increasing SI at the time of pre-hospital care in patients with a high baseline SI was associated with increased mortality [5, 9, 10]. The utilization of the SI as a dynamic variable makes sense, as it is straightforward, easy to comprehend and logical. A natural assumption might be that a longer delay to SI measurement would equate to an elevated SI, as more time has elapsed since the initial injury. However, this assumption did not hold true in our study. Thus, the dynamic nature of the SI seems to be limited. This finding is clearly in contrast to that of Nordin et al., who evaluated the paediatric age-adjusted SI as a dynamic variable from the pre-hospital setting to the emergency department and found significant association between the change of SI and outcome. However, in our study the time of measurement of the SIA/G was upon arrival of the HEMS unit. As such, the SI may be artificially enhanced by potential first-line, life-saving treatments provided by EMS units prior to HEMS arrival.

We found the discriminative ability of SIA/G regarding 30-day mortality in critically injured patients to be relatively high. Kimura et al. previously compared the discriminative ability of different variations of the SI and concluded that the SIA/G or rSIG/A is superior to the SI alone in trauma patients aged 55 years or older [4]. Earlier studies that integrated both age and the GCS into the SI reported that age is a major factor affecting mortality of severely injured trauma patients, with older age corresponding to higher mortality [5, 6]. Other studies demonstrated that the GCS, which has been widely adopted and readily describes the level of consciousness of a patient, is independently and strongly correlated with mortality among patients with a TBI [20, 21].

In the current study, the patients with the longest delay in measurement of the SI (≥ 40 min) had a lower GCS, more frequent advanced airway management and higher mortality. We found no difference in the incidence of TBI among the different time measurement delay groups, which would have conveniently explained the higher need for airway management and increased mortality in the group with the longest delay to SI measurement. A delayed time of arrival of a HEMS unit might indicate a longer distance, which, in turn, might lower the threshold for airway management. However, the differences in treatment and outcome of the patients between the delay groups are most likely explained by selection, as a HEMS mission can be cancelled by a HEMS physician if EMS personnel attending the scene report no need for interventions. In contrast, there might not be sufficient time to cancel a HEMS rescue mission if a HEMS unit arrives promptly at the scene.

The calculation of the original SI does not require complicated multiple variable inputs or difficult equations, making it a simple and useful tool in both pre-hospital and hospital settings. Although the addition of the GCS and age to the basic SI equation slightly increases the complexity of the calculation, its enhanced accuracy in predicting mortality is considerable when compared to the SI alone [4]. Furthermore, in an age of increasing automation and data-driven analytics, the incorporation of a mechanically calculated SI into EMS systems could provide a valuable tool in the pre-hospital setting. The potential benefits of an automatically calculated SI include the possibility of resource assessment based on the probability of mortality. In this way, EMS units might bypass a lower-level trauma centre and instead head directly to a level 1 trauma centre, thus reducing delays and unnecessary stopovers.

### Strengths and limitations

The national HEMS registry constituted a considerable strength of the study, as it covers all HEMS missions in the country. However, the patient population consisted of critically injured patients treated by a single HEMS unit. Thus, the generalizability of the findings to the general pre-hospital patient population is somewhat limited. Further confounding factors that may have affected the accuracy of the study are related to the recording of the data in the registries. The actual times from injury occurrence to injury logging and emergency service dispatch may not be entirely accurate, as delays may have arisen in contacting emergency commination centre. In addition, chronic illnesses or potentially decreased functional ability that the patients may have had prior to their injuries were not considered. The determination of the three groups regarding time-of-delay was made based upon geographically realities, relatively short delay times and the presumption that adequate differences with regard to mortality would be found between the groups.

## Conclusion

The discriminative ability of the SIA/G regarding 30-day mortality is not significantly affected by the time at which it is first measured during pre-hospital setting. The SIA/G is a simple and reliable tool for assessing the risk of 30-day mortality among severely injured patients in the pre-hospital setting.

## Supplementary Information

**Additional file 1. **

**Additional file 2. **

**Additional file 3. **

## Data Availability

The dataset used and analysed in the current study are available from the corresponding author on reasonable request.

## Abbreviations

AUROC: Area under the receiver operator characteristic curve
CIs: Confidence intervals
EMS: Emergency medical services
GCS: Glasgow Coma Score
HEMS: Helicopter emergency medical services
ROC: Receiver operator characteristic curve
rSI: Reverse shock index
SI: Shock index
SIA/G: Shock index multiplied by age and divided by the GCS
STROBE: Strengthening the Reporting of Observational Studies in Epidemiology
TBI: Traumatic brain injury
