# Supplementary material for: The effect of time of measurement on the discriminant ability for mortality in trauma of a pre-hospital shock index multiplied by age and divided by the Glasgow Coma Score: a registry study
Source: BMC Emerg Med. 2022 Nov 30;22:189. doi: 10.1186/s12873-022-00749-8 (PMC9710012; doi:10.1186/s12873-022-00749-8)
Supplement: Supplementary file 1 — Additional file 1. [file 12873_2022_749_MOESM1_ESM.docx]

Appendix 1/Supplemental digital content

*Appendix 1*ICD-10 codes included in the study. A *) denotes patients included in the traumatic brain injury group. All other included ICD-10 (#) codes were regarded as trauma. Patients in the trauma without traumatic brain injury only had a #) diagnosed; isolated traumatic brain injury had only a *) diagnosed, while patients with trauma and traumatic brain injury had a *) and #) diagnosed.

S06.4, Traumatic epidural hemorrhage, *

S06.5, Traumatic subdural hemorrhage, *

S06.6, Traumatic subarachnoidal hemorrhage, *

S06.1, Traumatic cerebral edema, *

S06.2, Diffuse traumatic brain injury, *

S06.3, Focal traumatic brain injury, *

S06.7, Intracranial injury with prolonged coma, *

S06.8, Other specified intracranial injuries, *

S06.9, Unspecified intracranial injury, *

S06.0, Concussion, *

J939., Pneumothorax, unspecified, #

J942., Hemothorax, #

S01.0, Open wound of scalp, #

S02, Fracture of skull and facial bones, #

S02.00, Fracture of vault of skull, closed, #

S02.01, Fracture of vault of skull, open, #

S02.1, Fracture of base of skull , #

S02.10, Fracture of base of skull without intracranial injury, #

S02.11, Fracture of base of skull with intracranial injury, #

S02.20, Fracture of nasal bones, closed, #

S02.21, Fracture of nasal bones, open, #

S02.30, Fracture of orbital floor, open, #

S02.31, Fracture of orbital floor, closed, #

S02.40, Fracture of malar and maxillary bones, closed, #

S02.41, Fracture of malar and maxillary bones, open, #

S02.47, Multiple fractures of malar and maxillary bones, #

S02.60, Fracture of mandible, #

S02.61, Fracture of condylar process of mandible, #

S02.62, Fracture of subcondylar process of mandible, #

S02.67, Multiple fractures of mandible, #

S02.69, Unspecified fracture of mandible, #

S02.7, Multiple fractures involving skull and facial bones, #

S02.70, Multiple fractures involving skull and facial bones, closed, #

S02.71, Multiple fractures involving skull and facial bones, open, #

S02.8, Fractures of other skull and facial bones, #

S02.80, Fractures of other skull and facial bones, closed, #

S02.81, Fractures of other skull and facial bones, open, #

S02.90, Fracture of skull and facial bones, part unspecified, closed, #

S02.91, Fracture of skull and facial bones, part unspecified, open, #

S03.3, Dislocation of other and unspecified parts of head, #

S03.5, Sprain and strain of joints and ligaments of other and unspecified parts of head, #

S05.1, Contusion of eyeball and orbital tissues, #

S07.0, Crushing injury of face, #

S07.1, Crushing injury of face, #

S07.8, Crushing injury of other parts of head, #

S07.9, Crushing injury of head, part unspecified, #

S08.0, Avulsion of scalp, #

S08.8, Traumatic amputation of other parts of head, #

S09.0, Injury of blood vessels of head, not elsewhere classified, #

S09.7, Multiple injuries of head, #

S09.8, Other specified injuries of head, #

S09.9, Unspecified injury of head, #

S12.2, Fracture of other specified cervical vertebra, #

S12.7, Multiple fractures of cervical spine, #

S12.8, Fracture of other parts of neck, #

S12.9, Fracture of neck, part unspecified, #

S13.0, Traumatic rupture of cervical intervertebral disc, #

S14.0, Concussion and oedema of cervical spinal cord, #

S14.1, Other and unspecified injuries of cervical spinal cord, #

S15.0, Injury of carotid artery, #

S15.1, Injury of vertebral artery, #

S15.7, Injury of multiple blood vessels at neck level, #

S15.9, Injury of unspecified blood vessel at neck level, #

S17.8, Crushing injury of other parts of neck, #

S19.8, Other specified injuries of neck, #

S19.9, Unspecified injury of neck, #

S22.0, Fracture of thoracic vertebra, #

S22.1, Multiple fractures of thoracic spine, #

S22.2, Fracture of sternum, #

S22.3, Fracture of rib, #

S22.4, Multiple fractures of rib, #

S22.5, Flail chest, #

S22.8, Fracture of other parts of bony thorax, #

S22.9, Fracture of bony thorax, parts unspecified, #

S23.3, Sprain and strain of thoracic spine, #

S24.0, Concussion and oedema of thoracic spinal cord, #

S24.1, Other and unspecified injuries of thoracic spinal cord, #

S25.0, Injury of thoracic aorta, #

S25.1, Injury of innominate or subclavian artery, #

S25.5, Injury of innominate or subclavian artery, #

S25.9, Injury of unspecified blood vessel of thorax, #

S26.0, Injury of heart with hemopericardium, #

S26.8, Other injuries of heart, #

S26.9, Injury of heart, unspecified, #

S27.0, Traumatic pneumothorax, #

S27.1, Traumatic hemothorax, #

S27.2, Traumatic hemopneumothorax, #

S27.3, Other injuries of lung, #

S27.6, Injury of pleura, #

S27.7, Multiple injuries of intrathoracic organs, #

S27.8, Injury of other specified intrathoracic organs, #

S27.9, Injury of unspecified intrathoracic organ, #

S28.0, Crushed chest, #

S29.0, Injury of muscle and tendon at thorax level, #

S29.7, Multiple injuries of thorax, #

S29.9, Unspecified injury of thorax, #

S32.0, Fracture of lumbar vertebra, #

S32.1, Fracture of sacrum, #

S32.2, Fracture of coccyx, #

S32.3, Fracture of Ilium, #

S32.4, Fracture of acetabulum, #

S32.5, Fracture of pubis, #

S32.7, Multiple fractures of lumbar spine and pelvis, #

S32.8, Fracture of other and unspecified parts of lumbar spine and pelvis, #

S33.0, Traumatic rupture of lumbar intervertebral disc, #

S33.4, Traumatic rupture of symphysis pubis, #

S33.7, Sprain and strain of other and unspecified parts of lumbar spine and pelvis, #

S34.0, Pelvis level concussion and oedema of lumbar spinal cord, #

S35.0, Injury of abdominal aorta, #

S35.1, Injury of inferior vena cava, #

S35.2, Injury of coeliac or mesenteric artery, #

S35.3, Injury of portal or splenic vein, #

S35.4, Injury of renal blood vessels, #

S35.7, Injury of multiple blood vessels at abdomen, lower back and pelvis level, #

S35.8, Injury of other blood vessels at abdomen, lower back and pelvis level, #

S35.9, Injury of unspecified blood vessel at abdomen, lower back and pelvis level, #

S36.0, Injury of spleen, #

S36.1, Injury of liver or gallbladder, #

S36.2, Injury of pancreas, #

S36.3, Injury of stomach, #

S36.4, Injury of small intestine, #

S36.5, Injury of colon, #

S36.6, Injury of rectum, #

S36.7, Injury of multiple intra-abdominal organs, #

S36.8, Injury of other intra-abdominal organs, #

S36.9, Injury of unspecified intra-abdominal organ, #

S37.0, Injury of kidney, #

S37.1, Injury of ureter, #

S37.2, Injury of bladder, #

S37.6, Injury of uterus, #

S39.0, Injury of muscle and tendon of abdomen, lower back, and pelvis, #

S39.7, Other multiple injuries of abdomen, lower back, and pelvis, #

S39.8, Other specified injuries of abdomen, lower back, and pelvis, #

S40.9, Superficial injury of shoulder and upper arm, unspecified, #

S42.0, Fracture of clavicle, #

S42.1, Fracture of scapula, #

S42.2, Fracture of upper end of humerus, #

S42.3, Fracture of shaft of humerus, #

S42.4, Fracture of lower end of humerus, #

S42.7, Multiple fractures of clavicle, scapula, and humerus, #

S42.8, Fracture of other parts of shoulder and upper arm, #

S44.7, Injury of multiple nerves at shoulder and upper arm level, #

S45.1, Injury of brachial artery, #

S45.9, Injury of unspecified blood vessel at shoulder and upper arm level, #

S47, Crushing injury of shoulder and upper arm, #

S48.1, Traumatic amputation at level between shoulder and elbow, #

S48.9, Traumatic amputation of shoulder and upper arm, level unspecified, #

S49.7, Multiple injuries of shoulder and upper arm, #

S49.9, Unspecified injury of shoulder and upper arm, #

S52.0, Fracture of upper end of ulna, #

S52.1, Fracture of upper end of radius, #

S52.2, Fracture of shaft of ulna, #

S52.3, Fracture of shaft of radius, #

S52.4, Fracture of shafts of both ulna and radius, #

S52.5, Fracture of lower end of radius, #

S52.6, Fracture of lower end of both ulna and radius, #

S52.7, Multiple fractures of forearm, #

S52.8, Fracture of other parts of forearm, #

S52.9, Fracture of forearm, part unspecified, #

S57.0, Crushing injury of elbow, #

S57.8, Crushing injury of other parts of forearm, #

S57.9, Crushing injury of forearm, part unspecified, #

S58.0, Traumatic amputation at elbow level, #

S58.1, Traumatic amputation at level between elbow and wrist, #

S58.9, Traumatic amputation of forearm, level unspecified, #

S59.9, Unspecified injury of forearm, #

S62.1, Fracture of other carpal bone(s), #

S62.2, Fracture of first metacarpal bone, #

S62.3, Fracture of other metacarpal bone, #

S62.4, Multiple fractures of metacarpal bones, #

S62.5, Fracture of thumb, #

S62.6, Fracture of other finger, #

S62.7, Multiple fractures of fingers, #

S68.4, Traumatic amputation of hand at wrist level, #

S68.8, Traumatic amputation of other parts of wrist and hand, #

S68.9, Traumatic amputation of wrist and hand, level unspecified, #

S69.7, Multiple injuries of wrist and hand, #

S69.8, Other specified injuries of wrist and hand, #

S72, Fracture of femur, #

S72.3, Fracture of shaft of femur, #

S72.4, Fracture of lower end of femur, #

S72.7, Multiple fractures of femur, #

S72.8, Fractures of other parts of femur, #

S72.9, Fracture of femur, part unspecified, #

S75.0, Injury of femoral artery, #

S75.7, Injury of multiple blood vessels at hip and thigh level, #

S75.8, Injury of other blood vessels at hip and thigh level, #

S75.9, Injury of unspecified blood vessel at hip and thigh level, #

S77.0, Crushing injury of hip, #

S77.1, Crushing injury of thigh, #

S77.2, Crushing injury of hip with thigh, #

S78.9, Traumatic amputation of hip and thigh, level unspecified, #

S79.7, Multiple injuries of hip and thigh, #

S8.1, Fracture of upper end of tibia, #

S8.22, Fracture of shaft of tibia, #

S8.23, Fracture of lower end of tibia, #

S82.4, Fracture of lower end of tibia, #

S82.5, Fracture of medial malleolus, #

S82.6, Fracture of lateral malleolus, #

S82.7, Multiple fractures of lower leg, #

S83.7, Injury to multiple structures of knee, #

S85.1, Injury of (anterior)(posterior) tibial artery, #

S85.7, Injury of multiple blood vessels at lower leg level, #

S87.0, Crushing injury of knee, #

S87.8, Crushing injury of other and unspecified parts of lower leg, #

S88.1, Traumatic amputation at level between knee and ankle, #

S88.9, Traumatic amputation of lower leg, level unspecified, #

S89.9, Unspecified injury of lower leg, #

S92.0, Fracture of calcaneus, #

S92.1, Fracture of talus, #

S92.2, Fracture of other tarsal bone(s), #

S92.3, Fracture of metatarsal bone, #

S92.7, Multiple fractures of foot, #

S93.0, Dislocation of ankle joint, #

S97.8, Crushing injury of other parts of ankle and foot, #

S98.3, Traumatic amputation of other parts of foot, #

S98.4, Traumatic amputation of foot, level unspecified, #

T04.3, Crushing injuries involving multiple regions of lower limb(s), #

T06.8, Other specified injuries involving multiple body regions, #

T07, Unspecified multiple injuries, #

T08, Fracture of spine, level unspecified, #

T09.3, Injury of spinal cord, level unspecified, #

T09.8, Other specified injuries of trunk, level unspecified, #

T14.5, Injury of blood vessel(s) of unspecified body region, #

T79.4, Traumatic shock, #
