# Supplementary material for: The effect of time of measurement on the discriminant ability for mortality in trauma of a pre-hospital shock index multiplied by age and divided by the Glasgow Coma Score: a registry study
Source: BMC Emerg Med. 2022 Nov 30;22:189. doi: 10.1186/s12873-022-00749-8 (PMC9710012; doi:10.1186/s12873-022-00749-8)
Supplement: Supplementary file 2 — Additional file 2. [file 12873_2022_749_MOESM2_ESM.docx]

**Appendix 2.** Z scores and P values for comparison of ROCs between time delay groups and patient groups.

| **Comparisons** |  | **Z** | **p** |
| --- | --- | --- | --- |
| All patients | Major trauma w/o TBI | 1,462 | 0,144 |
| All patients | Isolated TBI | 3,111 | 0,002 |
| All patients | Major trauma with TBI | 0,670 | 0,503 |
| Major trauma w/o TBI | Isolated TBI | 0,152 | 0,879 |
| Major trauma w/o TBI | Major trauma with TBI | 0,241 | 0,810 |
| Isolated TBI | Major trauma with TBI | 0,375 | 0,708 |
|  |  |  |  |
| All patients 0-19 min | All patients 20-39 min | 0,842 | 0,400 |
| All patients 0-19 min | All patients >40 | 1,013 | 0,311 |
| All patients 20-39 min | All patients >40 | 0,312 | 0,755 |
|  |  |  |  |
| Major trauma w/o TBI 0-19 min | Major trauma w/o TBI 20-39 min | 0,290 | 0,772 |
| Major trauma w/o TBI 0-19 min | Major trauma w/o TBI >40 min | 1,203 | 0,229 |
| Major trauma w/o TBI 20-39 min | Major trauma w/o TBI >40 min | 1,221 | 0,222 |
|  |  |  |  |
| Isolated TBI 0-19 min | Isolated TBI 20-39 min | 0,524 | 0,600 |
| Isolated TBI 0-19 min | Isolated TBI >40 min | 1,905 | 0,057 |
| Isolated TBI 20-39 min | Isolated TBI >40 min | 1,486 | 0,137 |
|  |  |  |  |
| Major trauma with TBI 0-19 min | Major trauma with TBI 20-39 min | 1,643 | 0,100 |
| Major trauma with TBI 0-19 min | Major trauma with TBI >40 min | 1,890 | 0,059 |
| Major trauma with TBI 20-39 min | Major trauma with TBI >40 min | 0,181 | 0,856 |
