# Supplementary material for: The effect of time of measurement on the discriminant ability for mortality in trauma of a pre-hospital shock index multiplied by age and divided by the Glasgow Coma Score: a registry study
Source: BMC Emerg Med. 2022 Nov 30;22:189. doi: 10.1186/s12873-022-00749-8 (PMC9710012; doi:10.1186/s12873-022-00749-8)
Supplement: Supplementary file 3 — Additional file 3. [file 12873_2022_749_MOESM3_ESM.docx]

Appendix 3.

Areas under receiver operating curves (95% confidence intervals).

|  | 0–19 minutes | 20–39 minutes | ≥40 minutes | All delay groups |
| --- | --- | --- | --- | --- |
| All patients | 0.88 (0.85–0.91) | 0.86 (0.84–0.89) | 0.86 (0.82–0.89) | 0.87 (0.85–0.89) |
| Trauma without TBI | 0.80 (0.68–0.91) | 0.82 (0.75–0.91) | 0.89 (0.79–0.99) | 0.83 (0.78–0.88) |
| Polytrauma with TBI | 0.94 (0.88–1.00) | 0.73 (0.49–0.97) | 0.76 (0.58–0.94) | 0.84 (0.76–0.92) |
| Isolated TBI | 0.84 (0.81–0.88) | 0.83 (0.79–0.87) | 0.78 (0.72–0.83) | 0.82 (0.80–0.85) |
